# Improving understanding and utilization of the antibiogram among medical residents

**DOI:** 10.1017/ash.2022.275

**Published:** 2022-08-18

**Authors:** Stephen J. Cooper, Christopher J. Destache, Renuga Vivekanandan

**Affiliations:** 1 Creighton University School of Medicine, Division of Internal Medicine, Omaha, Nebraska; 2 Creighton University School of Medicine, Division of Infectious Diseases, Omaha, Nebraska; 3 Creighton University School of Pharmacy and Health Professions, Omaha, Nebraska

## Abstract

In this quality improvement project, we sought to increase the understanding and utilization of the antibiogram among physicians in family medicine, internal medicine, and surgery residency programs at a Midwest Academic Healthcare institution. Through simple, inexpensive measures the comfort with, access to, and utilization of the antibiogram can be improved.

The antibiogram is a powerful tool for providers when selecting empiric antibiotic coverage. Its use is recommended by the Infectious Disease Society of America, the Centers for Disease Control and Prevention, and clinical decision support resources to aid in antibiotic stewardship and patient care.^1–3^ Antibiograms are a key component of antimicrobial stewardship programs, which have been shown to improve patient outcomes and shorten time to appropriate antimicrobial coverage.^4,5^ In previous studies, ∼30% of prescribers lacked confidence selecting empiric therapy using antibiogram data.^6^ Likewise, surveys have demonstrated that many residents are not comfortable using antibiograms as part of treatment decisions.^7^ Educating early trainees on antibiogram utilization and accessibility can provide guidance and confidence in their use as well as improve antimicrobial prescribing among practitioners.^5^

A 9-question survey was developed to determine resident physicians’ understanding regarding what an antibiogram is, as well as to assess their past exposure to infectious disease rotations, knowledge of how to access the institutional antibiograms, past use of antibiogram in patient care, and comfort level with using antibiograms (Fig. 1). This quality improvement project was reviewed and approved by the Creighton University Institutional Review Board. The survey was administered to every postgraduate year 1 (PGY1) resident in Creighton University’s family medicine, internal medicine, and surgery residency programs in April 2021 during the standard track’s tenth month of residency training. An educational poster explaining the proper function and use of the antibiogram was created (Fig. 2). The posters contained quick response codes that linked users to the institution’s antibiograms. Posters were placed in all family medicine, internal medicine, and surgery team rooms at the main teaching hospital for the Creighton University residency programs in July 2021. Lectures were given to each residency program during their weekly academic time in the first 2 months of the standard track year, July–August 2021. Then in January 2022 during the seventh month of the standard track year the same 9-question survey was readministered to the now postgraduate year 2 residents in Creighton University’s family medicine, internal medicine, and surgery residency programs as well as to the new PGY1 residents in the respective programs.

**Fig. 1.**
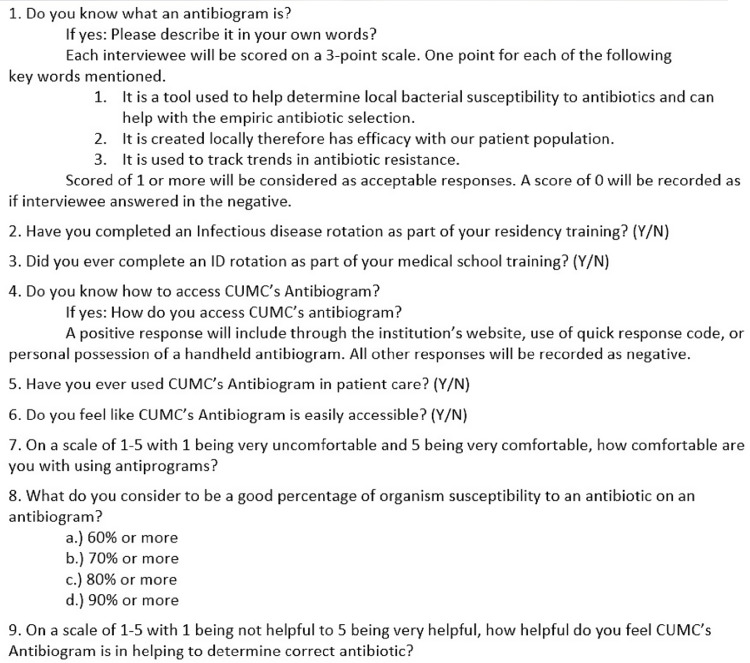
Nine-question survey.

**Fig. 2.**
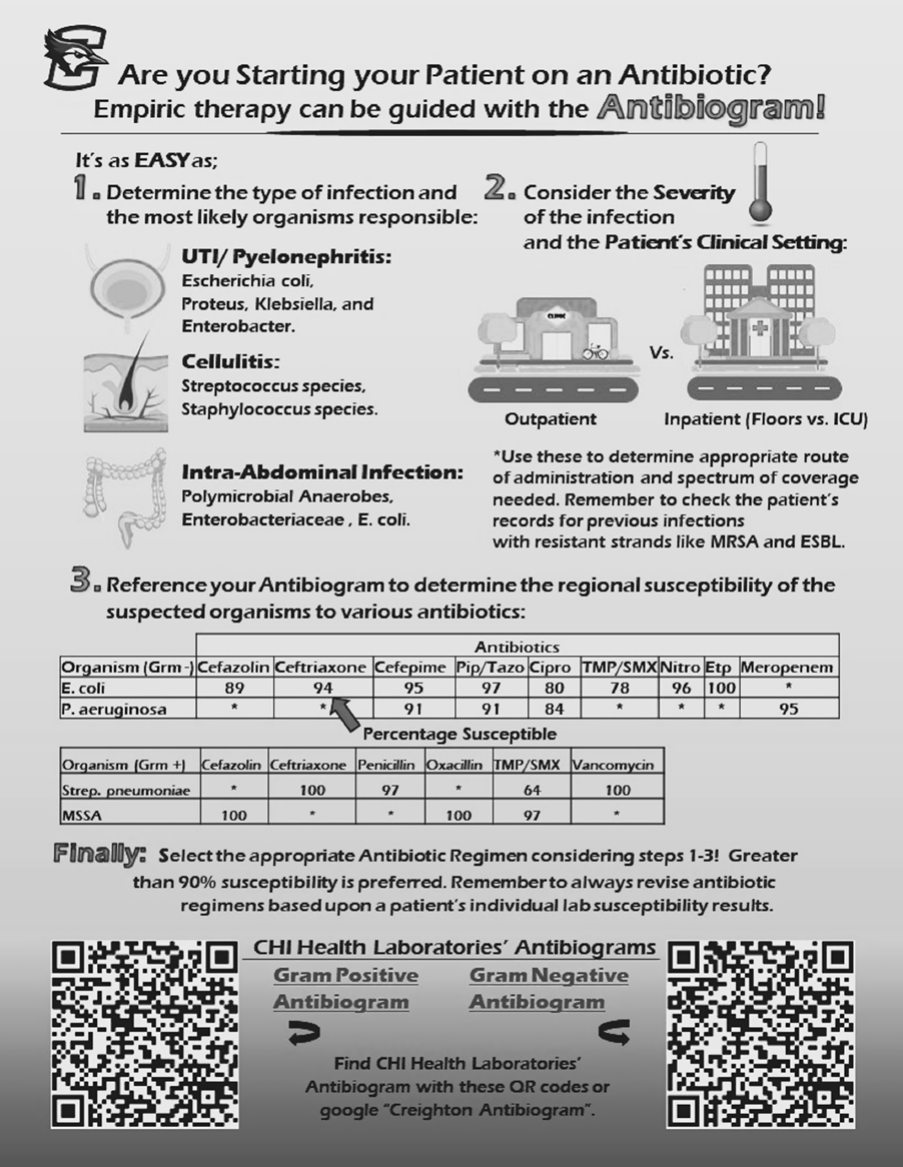
Educational poster displayed in team rooms.

All interviews were conducted over the telephone or in person by the same interviewer (S.C.). Questions were standardized to reduce bias. Both questions 1 and 4 had follow-up questions based on the interviewee’s response. This allowed for proper evaluation regarding their knowledge of antibiograms and how to access the institution’s antibiogram. Data were entered into an Excel spreadsheet (Microsoft, Redmond, WA) and were then transferred to SPSS for statistical analysis. Discrete variables were analyzed using the χ^2^ test or the Fisher exact test, and the Kruskal-Wallis test was used if there were >2 dependent variables. Continuous variables were analyzed using the Student *t* test as appropriate. Statistical significance was defined as *P* ≤ .05.

In total, 119 survey responses were obtained: 24 from family medicine, 79 from internal medicine, and 16 from surgery. Overall, 42 residents participated in 2021 and 77 participated in 2022, for a 100% response rate.

In total, 24 (57%) of 42 residents surveyed in 2021 and 69 (89%) of 77 residents surveyed in 2022 reported that they knew what an antibiogram was and were able to describe at least 1 attribute of it correctly. The increase of 32% was statistically significant (*P* < .001). Only 8 (21%) of 42 residents interviewed in 2021 could accurately describe a method for accessing the institution’s antibiogram. This significantly improved to 69 (90%) of 77 residents in 2022 (*P* < .001). Also, 16 residents (38%) reported using the antibiogram at least once in patient care in 2021, which significantly improved to 50 (65%) of the residents interviewed in 2022 (*P* < .001). Accessibility of the antibiogram was reported to be easy by 10 (24%) of 42 of residents in 2021, which improved to 60 residents (78%) in 2022 (*P* < .01). The average comfort level with using the antibiogram in 2021 was 3 on a scale of 1 to 5, which significantly increased to 3.78 in 2022 (*P* = .005). Assessing knowledge and the accurate use of the antibiogram was also determined in the survey. In total, 14 (33%) of 42 residents in 2021 and 41 (53%) of 77 residents in 2022 chose the correct answer (*P* > .05). On a scale of 1 to 5, the helpfulness of the antibiogram as a tool when trying to select empiric antibiotic choices averaged 3.64 in 2021, which significantly improved to 4.3 in 2022 (*P* = .015).

In addition, 14 residents in 2021 and 35 in 2022 reported completion of an infectious disease (ID) rotation in residency or medical school (*P* > .05). Residents who took the 2021 survey and reported prior infectious disease training in medical school or residency were compared to residents with no prior training. Significantly more residents with prior ID training [12 (86%) of 14] could define an antibiogram, compared to 12 (43%) of 28 residents with no prior ID training (*P* < .05). Similarly, more residents with prior ID training reported that they knew how to access the institution’s antibiogram [7 (50%) of 14 compared to 2 (7%) of 28] and had used it in patient care [9 (64%) of 14 compared to 7 (25%) of 28; *P* < .05]. The average comfort level with antibiograms was 4 for residents with prior ID training and 2.5 with no prior training on a scale of 1 to 5 (*P* < .05).

Our results verify a lack of exposure to antibiograms at the PGY1 residency level. This nescience is likely contributing to the discomfort reported by providers when selecting empiric antibiotic treatment regimens. Notably, the first survey was conducted in the tenth month of the standard track year. The second survey was conducted during the seventh month of the standard track year. Differences in results from 2021 compared to 2022 could have been due to total time spent in residency training. Nevertheless, PGY1 resident responses were significantly improved in 2022.

Our data analysis revealed a statistically significant increase in the number of residents who were able to define the antibiogram in 2022. This finding reflects the increased exposure to the antibiogram via educational posters and program specific lectures. Residents also reported being able to access the institution’s antibiogram at statistically higher rates in 2022, which is likely due to the placement of quick response codes on the educational posters that were placed in every team room. Residents with prior ID training in either medical school or residency reported significantly higher understanding of, comfort with, and utilization of the antibiogram than those without prior training.

Our quality improvement efforts show that simple and inexpensive interventions can increase providers’ understanding of, access to, comfort with, and utilization of antibiograms. All educational efforts were resident led. This peer-to-peer education method is nonthreatening and cost effective, benefiting our quality improvement efforts. The total cost of printing our posters was $37.99 USD. This was the only expense of our implementation efforts other than the time expended by the team. We recommend that other residency programs assess the use of and comfort with the antibiogram among their residents. We encourage the duplication of our education efforts and improvements to antibiogram accessibility. We also encourage others to explore new tactics to help improve trainee comfort with the antibiogram and empiric antibiotic regimen selection.
